# Error in Discussion

**DOI:** 10.1001/jamanetworkopen.2021.1376

**Published:** 2021-02-12

In the Original Investigation titled “Effect of Bimagrumab vs Placebo on Body Fat Mass Among Adults With Type 2 Diabetes and Obesity: A Phase 2 Randomized Clinical Trial,”^1^ published January 13, 2021, there were errors in the Discussion section. The penultimate sentence of the first paragraph should have referred to a 0.8% weight loss among patients receiving placebo. The final sentence should have read that weight loss with bimagrumab was greater than the 1-year FDA threshold of 5% for registration of new medicines for treating obesity and overweight. This article has been corrected.^1^

## References

[1] Heymsfield SB, Coleman LA, Miller R, Effect of bimagrumab vs placebo on body fat mass among adults with type 2 diabetes and obesity: a phase 2 randomized clinical trial. JAMA Netw Open. 2021;4(1):e2033457. doi:10.1001/jamanetworkopen.2020.33457 33439265 PMC7807292

